# A Comparison of the Capacity of Mesenchymal Stromal Cells for Cartilage Regeneration Depending on Collagen-Based Injectable Biomimetic Scaffold Type

**DOI:** 10.3390/life11080756

**Published:** 2021-07-27

**Authors:** Victor I. Sevastianov, Yulia B. Basok, Ludmila A. Kirsanova, Alexey M. Grigoriev, Alexandra D. Kirillova, Evgeniy A. Nemets, Anastasia M. Subbot, Sergey V. Gautier

**Affiliations:** 1 Department for Biomedical Technologies and Tissue Engineering, The Shumakov National Medical Research Center of Transplantology and Artificial Organs, 1 Shchukinskaja St., 123182 Moscow, Russia; bjb2005@mail.ru (Y.B.B.); ludochkakirsanova@mail.ru (L.A.K.); Bear-38@yandex.ru (A.M.G.); kirillovaad20@gmail.com (A.D.K.); evgnemets@yandex.ru (E.A.N.); gautier@list.ru (S.V.G.); 2 Laboratory of Fundamental Research in Ophtalmology, The Research Institute of Eye Diseases, 11A, B Rossolimo St., 119021 Moscow, Russia; kletkagb@gmail.com; 3 Department of Transplantology and Artificial Organs, Faculty of Medicine, The Sechenov University, 8-2 Trubetskaya St., 119991 Moscow, Russia

**Keywords:** mesenchymal stromal cells, articular cartilage, osteoarthrosis, collagen, hydrogel, decellularization

## Abstract

Mesenchymal stromal cells (MSCs) have shown a high potential for cartilage repair. Collagen-based scaffolds are used to deliver and retain cells at the site of cartilage damage. The aim of the work was a comparative analysis of the capacity of the MSCs from human adipose tissue to differentiate into chondrocytes in vitro and to stimulate the regeneration of articular cartilage in an experimental model of rabbit knee osteoarthrosis when cultured on microheterogenic collagen-based hydrogel (MCH) and the microparticles of decellularized porcine articular cartilage (DPC). The morphology of samples was evaluated using scanning electron microscopy and histological staining methods. On the surface of the DPC, the cells were distributed more uniformly than on the MCH surface. On day 28, the cells cultured on the DPC produced glycosaminoglycans more intensely compared to the MCH with the synthesis of collagen type II. However, in the experimental model of osteoarthrosis, the stimulation of the cartilage regeneration was more effective when the MSCs were administered to the MCH carrier. The present study demonstrates the way to regulate the action of the MSCs in the area of cartilage regeneration: the MCH is more conducive to stimulating cartilage repair by the MSCs, while the DPC is an inducer for a formation of a cartilage-like tissue by the MSCs in vitro.

## 1. Introduction

The major problem of healthcare in the industrial community is the damage of joint cartilage, which is associated with the limited capacity of the tissue to regenerate [1]. The gold standard in cell therapy of cartilage diseases today is the method of autologous chondrocyte implantation which has some disadvantages [2]. These include the traumatic biopsy of the healthy area of cartilage, the difficulty of expansion, and the possibility of cell dedifferentiation [3].

The application of autologous mesenchymal stem cells (MSCs) [4] may serve as an alternative therapeutic approach to the restoration of the cartilage tissue. The MSCs are multicomponent cells capable of differentiating into various types of cells, including chondrocytes, which allows us to use them in the process of creating cartilage-like structures as substitutes for damaged areas [5,6]. On the other hand, the therapeutic effect of the MSCs is based on paracrine secretion of a broad repertoire of growth factors and cytokines, which stimulate the proliferation of chondrocytes and the synthesis of the extracellular matrix (ECM) when injected into the joint [7].

For delivery and containment of the MSCs at the site of a cartilage defect, as well as in order to ensure vital activities of the cells over the time sufficient to trigger cartilage tissue repair processes, bioresorbable carriers (scaffolds or matrices) are used [8], which are mainly based on collagen [9]. Two types of collagen-based carriers, ECM biomimetics, are known that are suitable for minimally invasive intra-articular administration: a hydrogel scaffold derived from ECM components [10] and a finely dispersed scaffold of decellularized ECM [10,11,12,13]. The purpose of this work was to compare the effects of two types of microdispersed matrices on chondrogenic differentiation of the MSCs of human adipose tissue in vitro and on the processes of articular cartilage regeneration in an experimental model of rabbit knee osteoarthrosis (OA).

## 2. Materials and Methods

### 2.1. Obtaining and Culturing of MSCs

Adipose tissue samples weighing 3–5 g (*n* = 3) were obtained with the informed consent of living healthy donors during liver transplantation under general anesthesia. The study was conducted in accordance with the guidelines of the Helsinki Declaration and approved by the Local Ethics Committee at the Shumakov National Medical Research Center of Transplantology and Artificial Organs, Moscow, Russia (15 November 2019, Protocol No. 151119-1/1e). The tissue was incubated in 0.1% collagenase solution type I at 37 °C for 20 min. The MSCs were cultured in growth medium (DMEM/F12 (1:1)) with the addition of 10% fetal cattle serum, 100 U/mL penicillin, 100 μg/mL streptomycin sulfate, and 2 mM L-glutamine (all listed reagents—Gibco Inc, Billings, MT, USA). In the experiment, 3rd passage cells were used.

The immunophenotypic expression profile of cell markers isolated from the adipose tissue met the criteria of The International Society for Cellular Therapy and confirmed that these cells are multipotent MSCs [14]. In earlier studies, we found a high level of expression of CD29, CD44, CD49b, CD73, and CD90 in primary culture [15]. At the same time the expression of CD34, CD45, or HLA-DR was not observed in the culture [15].

### 2.2. Decellularization

Porcine femurs and knee joints were obtained at a slaughterhouse (OOO APK PROMAGRO, Russia) (one animal weighing about 120 kg). The tissues were decellularized according to the technique published earlier [16]. The cartilage was first cryo-ground in a cryo-mill (Retch GmBH, Haan, Germany). The number-weighted mean diameter of the cartilage microparticles did not exceed 220 μm, at the same time the particles with sizes 161 ± 11 μm prevailed. The decellularization included 3 “dry” freeze/thaw cycles (−196 °C/+37 °C). All subsequent steps were performed at room temperature under agitation (200 rpm) unless otherwise noted. The tissue was then subjected to three washes of sodium dodecyl sulfate (0.1% *w*/*v*) and Triton X-100 (1% *v*/*v*, 2% *v*/*v*, 3% *v*/*v* for the first, the second, and the third wash, respectively) for 72 h. Then the tissue was treated with 50 U/mL DNase type I (New England Biolabs Inc., Ipswich, MA, USA) for 48 h at 37 °C. After decellularization, the cartilage tissue was rinsed with deionized water with 100 U/mL penicillin (Gibco, Billings, MT, USA) and 100 μg/mL streptomycin sulfate (Gibco, Billings, MT, USA) for 72 h. Gamma radiation at a dose of 1.5 Mrad was used to sterilize the scaffold.

As a hydrogel ECM biomimetic, a microheterogenic collagen-based hydrogel (MCH) was selected. The injectable form of MCH (trademark Sphero^®^GEL, JSC Biomir service, Russia) registered in Russia for clinical use is produced from tissue ECM components of farm animals using the method of acetic acid extraction. The MCH scaffold consists of microparticles of collagen-based hydrogel crosslinked by gamma radiation and homogeneous hydrogel solution of the same composition at a ratio of 1:1. The main characteristics of MCH scaffolds are as follows: average size of microparticles—145.79 ± 0.09 μm; modulus of elasticity—1170 ± 12 Pa; viscosity module—62.9 ± 7.9 Pa; pH—7.0 ± 0.1; resorption time—up to 9 months. Water absorption is equal to 86.6 wt.%, while the content of bound water is at least 32.8 wt.%. It has been shown that the MSCs are able to form cartilage-like structures when cultured on the MCH [17].

### 2.3. Cell Seeding

The chondrogenic differentiation medium included high-glucose DMEM (Gibco, Billings, MT, USA), 10% ITS+ (Corning Inc, Corning, NY, USA), 1% sodium pyruvate (Sigma-Aldrich Corp, St. Louis, MO, USA), 0.25% ascorbate-2-phosphate (Sigma-Aldrich, St. Louis, MO, USA), 100 nM dexamethasone (Sigma-Aldrich, St. Louis, MO, USA), 0.002% TGF-β1 (PeproTech, Cranbury, NJ, USA), and 1% penicillin-streptomycin-glutamine (Gibco, Billings, MT, USA). The scaffolds (5 mg decellularized porcine articular cartilage (DPC) or 0.25 mL MCH) were seeded with 1 × 10^6^ MSCs by rotating in tubes with culture medium on the shaker Multi Bio 3D (BioSan, Riga, Latvia). The area of cartilage-like structures was measured on day 28 using ImageJ analysis software (National Institutes of Health, Washington, D.C, USA) for three representative samples.

The chondrogenic ability of MSCs cultured on scaffolds was compared with that in scaffold-free pellet culture, which served as a control. Chondrogenic media that contained 2 × 10^5^ MSCs were pipetted into individual wells of a 96-well V-bottom plate and centrifuged at 500× *g* for 5 min.

### 2.4. Calcein AM Staining

On day 28, the samples were stained with Calcein AM (Invitrogen Corp, Carlsbad, CA, USA), incubated for 30 min in the dark at 37 °C, and studied with a Nikon Ti microscope (Nikon Corp, Tokyo, Japan). The concentration of Calcein AM in the working solution was 2 × 10^−6^ M. Living cells were determined by green fluorescence (λ = 512 nm).

### 2.5. Histological Staining

The samples were fixed in formalin, washed in running water, and dehydrated in escalating concentrations of ethanol, kept in a mixture of ethanol and chloroform, then chloroform alone, and poured into paraffin. The sections were dewaxed, rehydrated, and stained following standard procedures, with hematoxylin and eosin, alcyan blue, and with Masson’s staining. The analysis and photography of the obtained preparations were carried out using a Nikon Eclipse microscope.

### 2.6. Immunohistochemical Staining

Collagen II in samples were visualized using Concentrated Peroxidase Detection System and antibodies to collagen II (all listed reagents—Novocastra, Leica Microsystems, GmbH, Wetzlar, Germany). Slides with paraffin sections (samples) were deparaffinized and in order to unmask an antigen were subjected to a preliminary enzymatic treatment. Thus, the samples were incubated for 5 min in distilled water at 37 °C, transferred to a Petri dish, the section was treated with a 1% trypsin solution on the Tris-buffer (TBS) for 45 min and rinsed with deionized water for 3 min. In order to neutralize endogenous peroxidase, the Peroxidase Block treatment was applied for 5 min, the samples were washed twice (5 min each) with TBS, and in order to prevent non-specific binding, incubated with Protein Block for 5 min. After double washing (5 min each) of the samples with TBS, primary antibodies were applied to the section (Leica rabbit antiCollagen 2-antibodies used with a 1:20 dilution) and the samples were incubated for 2 h at room temperature. Then the samples were washed twice with TBS (5 min each) with secondary antibodies introduced to the section for 30 min at room temperature (Biotinulated secondary antibody—goat-antimouse IgM, horse-antimouse IgG, horse-antirabbit IgG). After this, the samples were washed twice for 5 min with TBS, incubated with Streptavidin HRP during 30 min with the subsequent double TBS washing (5 min each) with the subsequent application of a dye substrate (DAB Working solution) to the section for 5 min. The samples were rinsed in distilled water, contrasted with hematoxylin, dehydrated, and placed in balsam. The stain results of the obtained histological samples were observed with a microscope.

### 2.7. Scanning Electron Microscopy (SEM) Preparation and Imaging

The morphology of the surface and the nearest subsurface layer of samples were examined with SEM using lanthanoid contrast [18]. The treatment was carried out with BioREE staining kit (LLC Glaucon, Moscow, Russia) in accordance with the manufacturer’s instructions and included primary flushing (for removal of sorbed growth medium), holding in contrasting solution (an isotonic saturated water solution of a salt of a rare earth metal), rinsing with distilled water, and removing the excessive moisture from the sample surface. Then the sample was placed on a microscope stage. The observations were made using EVO LS10 (Zeiss, Oberkochen, Germany) with a backscattered electron (BSE) detector in low vacuum mode (EP, 70 Pa), at accelerating voltage of 20 kV.

### 2.8. DNA Concentration Determination

DNA was isolated using the DNeasy Blood & Tissue Kit (QIAGEN, Hilden, Germany) according to the manufacturer’s instructions. The samples were digested with AL buffer and proteinase K for 16 h at 56 °C. DNA content was quantified using the ™ Picogreen Quant-iT (Invitrogen Corp, Carlsbad, CA, USA) according to the manufacturer’s instructions. Fluorescence was measured at 535 nm with excitation at 485 nm, and DNA content was quantified using a standard curve. Three samples from every cartilage-like structure were collected at each time point.

### 2.9. Glycosaminoglycans (GAG) Concentration Determination

The samples of culture medium (*n* = 5) were selected on days 7, 14, 21, and 28. Staining was performed in a 96-well plate, adding 20 µL of the sample and 200 μL of the 1.9- dimethylmethylene blue medium (Sigma-Aldrich, St. Louis, MO, USA) followed by determination on a Tecan Spark 10M spectrofluorimeter (Tecan Trading AG, Männedorf, Switzerland) at 525 nm wavelength.

### 2.10. Induction of OA

New Zealand White rabbits (males) were used in the experiments, weighing 3–4 kg (*n* = 17). The manipulations did not cause pain to the animals and were carried out in compliance with the Russian legislation: GOST 33215-2014 (Guidelines for accommodation and care of laboratory animals. Rules for equipment of premises and organization of procedures) and GOST 33216-2014 (Guidelines for accommodation and care of laboratory animals. Rules for the accommodation and care of laboratory rodents and rabbits). The work was approved by the Local Ethics Committee at the Shumakov National Medical Research Center of Transplantology and Artificial Organs, Moscow, Russia (January 24, 2020, Protocol No. 240120-1/1e). The study used the model of methylated bovine serum albumin antigen-induced of rabbit knee osteoarthritis evolving into OA [19] with modification scarification [20]. The choice of the OA model was determined by its low injury rate and high reproducibility [19], while a 2-month follow-up after OA was made based on previously conducted experiments [21].

All the animals were divided into 6 groups: 1 group (control) of intact rabbits (*n* = 2) and 5 groups of rabbits (*n* = 15) were used to create the OA model. On the 30th day after simulated OA, knee joints of the right hind paw of the rabbits of the experimental groups were injected with 1 × 10^6^ MSCs, 0.5 mL of MCH separately or with 1 × 10^6^ MSCs, 0.5 mL of the growth culture medium without serum with 5 mg DPC separately or with 1 × 10^6^ MSCs (the cells were mixed with the carrier 3 h before administration). A saline solution was injected into the knee joint of the left hind paw of the animals of each experimental group with OA. Before intra-articular administration of the MSCs, the rabbits were immunosuppressed with Sandimmun (active substance content of cyclosporin 50 mg/mL) in a dosage of 5 mg/kg. The duration of animal follow-up after sample administration was 2 months. To confirm the development of OA, hematological, radiological, and histological methods of investigation were carried out. In order to confirm the presence of arthrosis in rabbits on day 49 of the study, erythrocyte subsidence rate and white blood cell count were determined. The design of the experiment is presented in Table 1.

### 2.11. Statistical Analysis

The validity of the differences was determined using Student’s *t*-test (standard software package Microsoft Excel 2007). The differences were considered statistically significant if *p* < 0.05.

## 3. Results and Discussion

### 3.1. Chondrogenic Differentiation of MSCs

**Figure 1:**
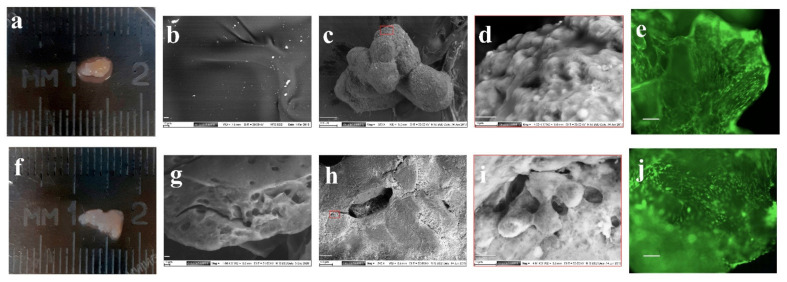
Morphological study of chondrogenic differentiation of the mesenchymal stromal cells (MSCs) on decellularized porcine cartilage (DPC) (**a**,**c**–**e**) and microheterogenic collagen-based hydrogel (MCH) scaffolds (**f**,**h**–**j**). Scanning electron microscopy (SEM) image of DPC (**b**) and MCH (**g**), (scale bar = 10 μm); SEM image (**c**,**h**) (scale bar = 100 μm); SEM image (**d**,**i**) (scale bar = 10 μm); calcein AM staining (**e**,**j**), (scale bar = 100 μm).

On micrographs (SEM analysis) the pattern surface structure of DPC is smooth (Figure 1b). The cells lack the lacunae characteristic of articular cartilage tissue.

The surface of the MCH sample appears flat and uniform (Figure 1g). Figure 1a,b shows the appearance of cartilage-like structures obtained on day 28 of culturing of MSCs with scaffolds in a differentiated chondrogenic medium. With the same initial number of MSCs cultured, the size of DPC-MSCs (Figure 1a) (area 19 ± 5 mm^2^) was higher than MCH–MSCs conglomerates (Figure 1f) (area 8 ± 3 mm^2^). The DPC and MCH microparticles are bonded by cells to form a single structure (Figure 1c,h) with cell formations on the surface of scaffolds (Figure 1d,i).

The use of lanthanoid contrast made it possible to visualize the nuclei and edges of the plasma membrane in some cells. Interestingly, the cytoplasm of cells included numerous granules resembling the DPC microparticles by color intensity. Intracellular vesicles can be associated with both secretion of extracellular cartilage matrix components and its resorption. When staining with Calcein AM, the adhesion and expansion of viable cells both on the DPC (Figure 1e) and the MCH surface (Figure 1j) were observed.

**Figure 2:**
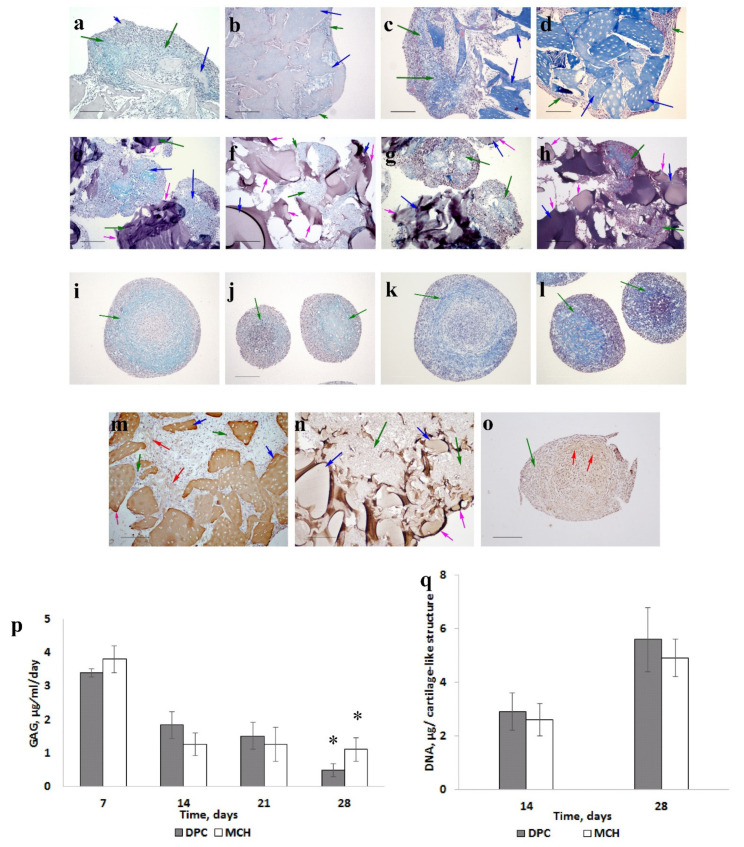
The dynamics of cartilage-like tissue formation (scale bar = 200 μm). MSCs cultured on DPC (**a**–**c**,**d**,**m)**; MSCs cultured on MCH (**e**–**h**,**n**); MSCs pellet culture (**i**–**l**,**o)**. Fourteen days (**a**,**c**,**e**,**g**,**i**,**k)**; twenty-eight days (**b**,**d**,**f**,**h**,**j**,**l)**. Alcian blue staining (**a**,**b**,**e**,**f**,**j**,**l)**; Masson staining (**c**,**d**,**g**,**h**,**k**,**l)**; collagen type II staining (**m**–**o**). Green arrows—cells with extracellular matrix, blue arrows—scaffolds, red arrows—collagen type II, and pink arrows—scaffold surface areas without cells. Glycosaminoglycans being released into the culture medium (**p**). * — p <0.05. DNA content (**q**).

On the 14th day of culturing of the MSCs, local positive staining was observed in cell-synthesized ECM in the DPC and MCH samples on GAG and collagen (Figure 2a,c,e,g).

On the 28th day, uniform staining on GAG (Figure 2b), and the presence of type II collagen (Figure 2m) were detected only in samples with DPC.

In the control sample we also observed chondrogenic differentiation of MSCs: the ECM contained GAG (Figure 2i,j) and collagen (Figure 2k,l), including type II collagen (Figure 2o). However, heterogeneity of the ECM was observed in pellets.

The level of GAG released into the media during 21 days of culturing was the same for both matrices (Figure 2p). However, the cartilage-like structure with DPC on the 28th day released less GAG into the culture medium compared to the sample with MCH (*p* < 0.05). It can be a positive factor for higher GAG accumulation, because most of the synthesized GAG will remain inside the structure. No significant difference in DNA content was observed between the two groups (Figure 2q).

The results obtained show that the MSCs are able to form cartilaginous structures when cultured in a chondrogenic differentiation medium with the MCH and DPC scaffolds. It can be assumed that chondrogenesis of the MSCs has been influenced by macromolecules such as hyaluronic acid, chondroitin sulfate, and type II collagen when cultured on the DPC (the MCH includes type I collagen) [22,23]. It has been shown that type II collagen, the main protein component of the ECM hyaline cartilage from which DPC was derived, can contribute to the preservation of chondrocyte morphology and the synthesis of more GAG than type I collagen [24]. Type II collagen is also known to enhance chondrogenic differentiation of MSCs when added to agarose scaffolds [25].

Note that the cells were distributed more evenly on the surface of DPC, whereas in case of MCH, the cells adhered and proliferated only on certain areas of the scaffold surface. This is probably due to the retention of site adhesion of the cells on the surface of DPC microparticles. Note that the advantage of decellularized tissue scaffolds of supporting the cells performing specific functions has been shown in a number of studies.

### 3.2. Stimulating Cartilage Regeneration in an Adjuvant Model of Rabbit Knee Osteoarthritis Evolving into Osteoarthrosis

It has been established that in all rabbits with simulated OA, hematological parameters were significantly higher than in intact animals (control): erythrocyte subsidence rate before OA 1.5 ± 0.3 mm/hour, after OA 3.0 ± 0.4 mm/hour, white blood cell count before OA (9.75 ± 0.5) × 10^9^/L, after (13.25 ± 0.9) × 10^9^/L.

**Figure 3:**
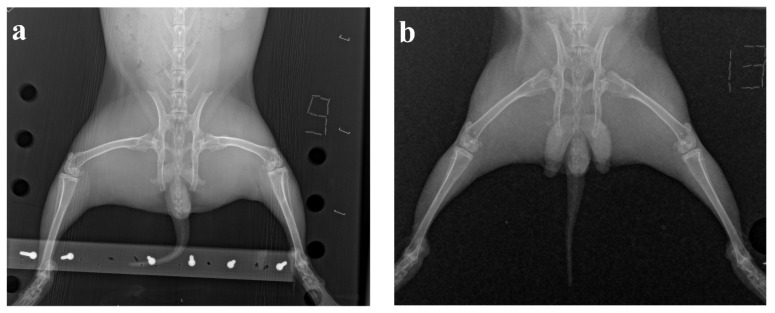
The results of X-ray examination: (**a**) osteoarthrosis model, (**b**) intact animal.

In X-rays, an uneven narrowing of the articular slit was found in the medial areas of the knee joints for rabbits with the model OA, which confirms the onset of OA on the 50th day after the first administration of methylated albumin (Figure 3).

At the end of the experiment, the intensity of the inflammatory process decreased, no significant difference between the knee joints in both groups of animals was visually found on the radiographs.

**Figure 4:**
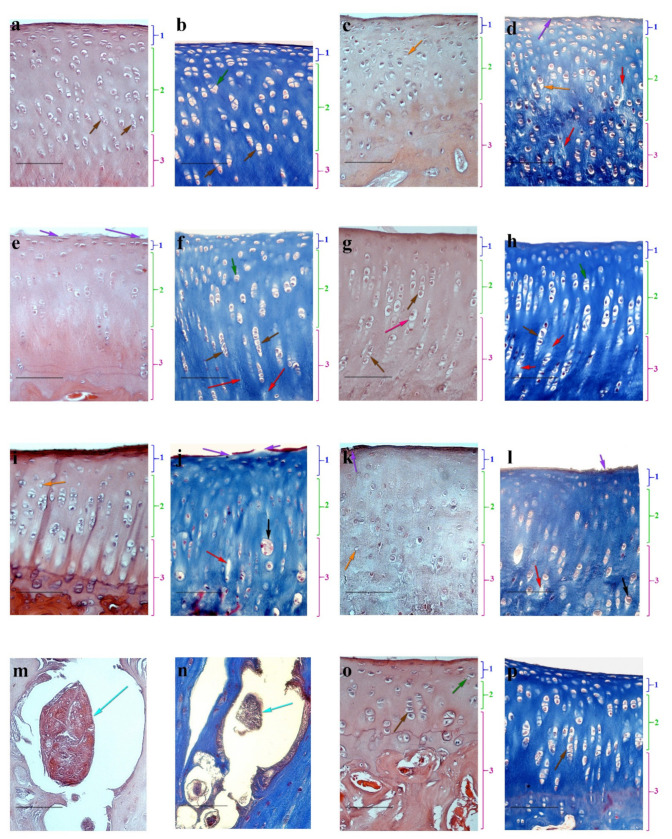
Haematoxylin-eosin (**a**,**c**,**e**,**g**,**i**,**k**,**m**,**o**) and Masson (**b**,**d**,**f**,**h**,**j**,**l**) staining of articular cartilage of rabbit knee, (scale bar = 100 μm). Intact animals (control group) (**a**,**b**); pathological section (control) (**c**,**d**); MSCs (**e**,**f**); DPC (**g**,**h**); MCH (**i**,**j**); MSCs + DPC (**k**–**n**); MSCs + MCH (**o**,**p**). 1—superficial layer, 2—intermediate zone, 3—basal layer. Orange arrows—empty lacunae; red arrows—unfolding of the ECM; purple arrows—ulcerations of the surface plate; green arrows—isogenous groups; pink arrows—hypertrophied chondrocytes; grey arrows—clusters; blue arrows—independent dense structures; brown arrows—vertical columns.

The most pronounced morphological changes in cartilage structure for all animal experimental groups were observed in the articular cartilage of the tibia. In the healthy articular cartilage of intact animals (*n* = 2), superficial, intermediate, and basal layers were clearly visible. In the surface layer, an acellular plate was visualized, with elongated flattened and oval cells, chondroblasts, and young chondrocytes underneath. In the middle layer in the eosinophilic fine-fibered ECM, chondrocytes often form vertical columns characteristic of the articular cartilage, most noticeable in the large tibia. In the basal layer bordering the subchondral bone, rounded cartilage cells were detected (Figure 4a,b).

In all animals of the experimental groups (*n* = 15), the cartilage from the left knee joint of the tibia showed morphological signs of altered structure (Figure 4c,d). Ulcerations and leaks of the surface plate were observed. Destructurization of basal layers was expressed by depletion of the ECM cells, weak expression of the superficial layer, empty lacunae, disappearance of the column structure, chaotic arrangement of cartilage cells, swelling, and focal unfolding of the ECM. At the same time, the chondrocytes in samples looked hypertrophied.

On day 60, after intra-articular administration to animals, experimental groups of MSCs, DPC, or MCH showed signs of partial cartilage repair. A significant number of cells in the surface layer and the formation of column chondrocytes were determined upon administration of MSCs. However, for that group, partial damage to the surface plate, ulceration of the surface layer, and slight defibration of ECM remained (Figure 4e,f). This result can be associated with low cell viability at the site of administration when administered as a suspension. After the implantation into an experimental group of DPC animals, a pronounced middle layer comprising chondrocyte columns and a significant number of isogenous groups were observed in articular cartilage (Figure 4g,h). However, regeneration of damaged cartilage was not complete, as manifested by depletion of the surface layer cells and the presence of hypertrophied chondrocytes. In contrast, for an experimental group of animals with the introduction of MCH, the articular cartilage surface plate was clearly expressed, but the complete restoration of its integrity was not achieved. Note that clusters and stratification were present in the samples of ECM (Figure 4i,j), which refer to changes characteristic of damaged cartilage tissue [26].

When the MSCs on the DPC scaffold were injected into the damaged joint of animals with OA, we obtained an unexpected result. There were no signs of cartilage tissue regeneration (Figure 4k,l), and independent dense structures were found in the scarification sites that were not associated with articular cartilage, which included living and dead cells, ECM, and a surface layer separating them from the environment (Figure 4m,n). The presence of its own surface layer, which restricts the structure from the environment and hinders mass transfer, indicates that the cells are oriented towards the secretion of factors inside the conglomerate, and not into the joint cavity to stimulate cartilage regeneration. At present, we cannot explain the origin of the cells forming this structure. This issue requires additional research including the use of labeled cells.

After the introduction of MSCs on the MCH scaffold, signs of cartilage regeneration were detected (Figure 4o,p): an increase in the number of chondroblasts and young chondrocytes in the surface layer was registered relative to the control group. In the middle layer, the chondrocytes were arranged in columns. The appearance of isogenous groups was observed in the basal layer.

Apparently, the scaffold MCH induces the secretory activity of MSCs to a greater extent than the tissue-specific scaffold DPC, whereas DPC is a stimulator for the formation of cartilage-like structures. It can be assumed that the differences in the functional activity of DPC and MCH scaffolds are based on the dependence of cell behavior on the elasticity, topography, and chemical composition of the carrier [27,28,29]. Interestingly, in a comparative study of gelatin gels with different rigidity, it was demonstrated that the gel with high rigidity promoted osteogenic differentiation of MSCs yet polarized macrophages towards the M1 phenotype, which is characterized by the production of proinflammatory cytokines [30]. Wu et al. also demonstrated the advantage of the ECM-based gel over decellularized bone microparticles for tissue regeneration in the rat periodontal model [31]. At the same time, it was shown that microparticles induce macrophages to proinflammatory (M1) polarization, and the gel obtained from the ECM polarizes macrophages to the regulatory/anti-inflammatory (M2) phenotype [31]. In contrast, Westman et al. demonstrated that microparticles of decellularized tissues have the potential for cell delivery and paracrine therapy in the conditions of impaired regeneration [32]. A possible reason for the difference between the results obtained in this work and a number of results described in publications [32,33] may be the peculiarities of the mechanical properties and density of the studied tissues—in the examples, soft tissues are used, while this study used dense cartilage. Note that the effect of microparticles of cartilage in vivo on the stimulation of regeneration in degenerative diseases, such as OA, has not been previously studied, and only cases of implantation of MSCs with DPC in surgically created defects have been described [34,35].

## 4. Conclusions

The MCH and DPC scaffolds are able to support in vitro the adhesion, proliferation, and differentiation of MSCs in the chondrogenic direction. However, the OA model shows the absence of regeneration processes after injection of the MSCs on the DPC scaffold into the damaged joint against the background of swelling and focal unfolding of ECM. Ulcerations of the surface plate and depletion of cells were observed. On the contrary, upon the injection of the MSCs on the MCH scaffold, signs of cartilage regeneration are detected.

On the other hand, the DPC scaffold provides the formation of a collagen-like structure in vitro, characterized by a uniform distribution of cells and the ECM synthesized by them, containing GAG and collagen, including type II collagen, a specific component for hyaline cartilage. This may be useful for developing technologies for the formation (“culturing”) in vitro based on a tissue-specific matrix and autologous MSCs of cartilage-like tissue for reconstructive and plastic surgery.

Thus, the present study demonstrates the way to regulate the action of the MSCs in the area of cartilage regeneration: the MCH is more conducive to stimulating cartilage repair by the MSCs, while the DPC is an inducer for a formation of a cartilage-like tissue by the MSCs in vitro.

The presented in vitro and in vivo results for the MSCs on the MCH and DPC scaffolds allow us to hope for the future clinical application of this biomedical product for the restoration of damaged tissues of the musculoskeletal system (joints, ligaments, tendons, muscles, and bones).

## Figures and Tables

**Table 1:** The design of the experiment.

Days	−1	1	15	29	49	50	110	111
Group formation								
Subcutaneous administration of methylated bovine serum albumin (mBSA) and Freunds complete adjuvant								
Intra-articular injection of mBSA								
Immunosuppression								
Intra-articular injection of samples								
Blood test								
X-ray examination								
Euthanasia								

## Data Availability

The data presented in this study are available on request from the corresponding author.
